# High-quality AlN epitaxy on nano-patterned sapphire substrates prepared by nano-imprint lithography

**DOI:** 10.1038/srep35934

**Published:** 2016-11-04

**Authors:** Lisheng Zhang, Fujun Xu, Jiaming Wang, Chenguang He, Weiwei Guo, Mingxing Wang, Bowen Sheng, Lin Lu, Zhixin Qin, Xinqiang Wang, Bo Shen

**Affiliations:** 1 State Key Laboratory of Artificial Microstructure and Mesoscopic Physics, School of Physics, Peking University, Beijing 100871, China; 2 Anhui Key Laboratory of Detection Technology and Energy Saving Devices, Anhui Polytechnic University, Wuhu 241000, China; 3 Collaborative Innovation Center of Quantum Matter, Beijing 100871, China

## Abstract

We report epitaxial growth of AlN films with atomically flat surface on nano-patterned sapphire substrates (NPSS) prepared by nano-imprint lithography. The crystalline quality can be greatly improved by using the optimized 1-μm-period NPSS. The X-ray diffraction ω-scan full width at half maximum values for (0002) and (10
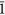
2) reflections are 171 and 205 arcsec, respectively. The optimized NPSS contribute to eliminating almost entirely the threading dislocations (TDs) originating from the AlN/sapphire interface via bending the dislocations by image force from the void sidewalls before coalescence. In addition, reducing the misorientations of the adjacent regions during coalescence adopting the low lateral growth rate is also essential for decreasing TDs in the upper AlN epilayer.

In recent years, heteroepitaxy of AlN template with high crystalline quality has attracted considerable attentions, owing to its great potential in fabrication of high performance AlGaN-based optoelectronic devices^1^,^2^. Generally, the surface morphology and defect density of AlN template have a crucial influence on the crystalline quality of AlGaN and upper active layer in deep ultraviolet (DUV) devices. Therefore, it is essential to obtain AlN template with flat surface and low threading dislocation density (TDD). Up to date, sapphire substrate is still the preferred choice for AlN heteroepitaxy due to its high transparency in the UV region and low cost. However, epitaxy of AlN template with low TDD remains a challenge due to the serious mismatch between AlN and sapphire in terms of lattice constant and thermal expansion coefficient. Moreover, due to the low surface mobility of Al adatoms caused by high diffusion barriers and very strong Al-N bonding^3^, it is hard to tailor the growth process for AlN as that for GaN adopting the “two-step” approach, which thus results in a very high TDD in AlN films, typically in the order of 10^9^–10^10^ cm^−2^. High TDD reduces the internal quantum efficiency (IQE) of DUV multiple quantum wells (MQWs) significantly because of nonradiative recombination of carriers at dislocations. In this case, the IQE of DUV MQWs, as a general rule, is at a low level^1^.

Many techniques have been proposed and made great progress in reducing TDD of AlN templates considering the above situation, such as middle-temperature AlN interlayer in metal-organic chemical vapor deposition (MOCVD)^2^ and migration-enhanced MOCVD (MEMOCVD)^4^. Besides, epitaxial lateral overgrowth (ELO) techniques on micro-stripe patterned sapphires or AlN/sapphire templates have also attracted much attention and significantly reduced the TDD^5^,^6^,^7^,^8^,^9^. However, large spacing between micro-patterns usually requires a great coalescence thickness close to 10 μm, implying long growth time and high cost. To obtain a thinner coalescence thickness, AlN epitaxy on nano-patterned sapphire substrates (NPSS) and nano-patterned AlN/sapphire template are attracting much attention, exhibiting great potential in high-quality AlN growth^10^,^11^. For AlN epitaxy on NPSS, although the outcome is exciting, the mechanism underlying the effect of the pattern on dislocation evolution and coalescence process has not been well investigated to the best of our knowledge, which is vital for reproducible fabrications of uniform and large-size AlN template. In this study, a rapid and stable method combined with nano-imprint lithography to prepare 2-in. uniform NPSS for AlN epitaxy is proposed, which is potential and convenient for stable and massive application according to our experiment result. Besides, the principle of defect control of AlN epitaxy on NPSS is investigated systematically by optimizing the patterns of NPSS and epitaxial conditions. Especially, the dependences of AlN crystalline quality on patterns of the NPSS and the lateral growth rate are discussed in detail.

## Results

**Figure 1:**
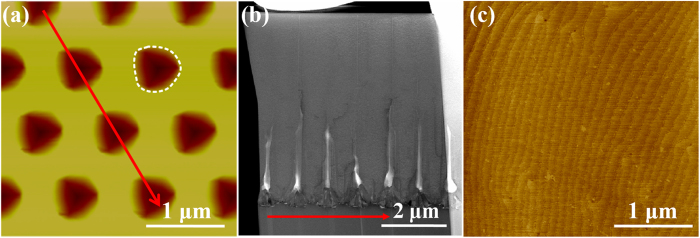
(**a**) AFM image of a typical NPSS (3 × 3 μm^2^). (**b**) The cross-sectional STEM image for this chosen sample fabricated by focused ion beam. (**c**) A typical AFM image of the surface morphology of the AlN sample on NPSS with 650-nm holes patterns (3 × 3 μm^2^).

Figure 1(a) shows a typical 3 × 3 μm^2^ AFM image of the prepared NPSS with hexagonal hole patterns in this work, and the fabrication process is demonstrated in the method section. The patterns on the NPSS are shaped as concave cones with a period of 1 μm, corresponding to the period of the nano-imprint stamp. The white dash line depicts the outline of the pattern on the surface. To facilitate the analysis, the outline is simplified as a circle with the diameter of D nm. Then the width of epitaxial mesa can be expressed as (1000-D) nm. NPSS with patterns in different sizes are named D/1000 NPSS. By changing the conditions of inductively coupled plasma (ICP) etching, the ingredients of corrosive liquid and the time of wet etching, different patterned substrates with hole diameters being about 300, 450, 650 and 800 nm were prepared. After that, the AlN films were grown on the above substrates under the same epitaxial conditions, including a 25-nm-thick low temperature nucleation layer and a 6-μm-thick high temperature AlN layer. Figure 1(b) displays the cross-sectional scan transmission electron microscope (STEM) image. The red arrow on the NPSS AFM image in Fig. 1(a) depicts the cross-sectional direction of substrate in Fig. 1(b). It is found that the AlN epilayer has completely coalesced in a thickness about 3 μm, which is less than the previously reported coalescence thickness for AlN grown on micro-stripe patterned substrates via ELO technique^4^,^12^. It should be ascribed to the nano-scale patterns on the substrate and the effective control of the AlN lateral growth. Figure 1(c) presents a typical 3 × 3 μm^2^ AFM image of the surface morphology for the as-grown AlN sample on 650/1000 NPSS. The image displays a flat surface with long, uniform and parallel atomic steps. Simultaneously, a few outcrops of dislocations are observed. The root mean square (RMS) roughness in the area of 3 × 3 μm^2^ is as small as 0.096 nm, indicating that atomically flat surface of the AlN films can be achieved on NPSS.

**Figure 2:**
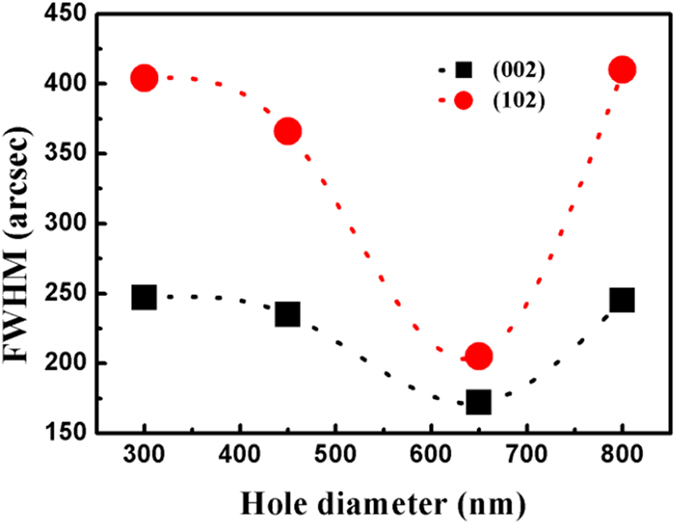
Dependence of the XRC FWHM of AlN epilayers on hole diameter of the NPSS.

Figure 2 illustrates the dependence of the X-ray rocking curves (XRCs) full width at half maximum (FWHM) values for the AlN symmetric (0002) and asymmetric (10
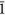
2) diffractions on the hole diameter of the NPSS. The dash lines are guide lines for eyes to catch the trend of these two data sets. It is clearly noticed that the FWHM value of the (0002) peak decreases from 247 to 171 arcsec as the hole diameter increases from 300 to 650 nm, and then the value increases to 245 arcsec when the diameter increases to 800 nm. The narrowest value 171 arcsec is realized on the 650/1000 NPSS. Comparably, the FWHM value of the asymmetric (10
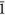
2) peak also presents a similar variation process, i.e., first decreasing from 404 to 205 arcsec, then increasing to 410 arcsec, where the narrowest FWHM value 205 arcsec is also realized on the 650/1000 NPSS. It implies that both the tilt and twist features of the grain structure for AlN can be greatly improved adopting the optimized hole diameter, about 650 nm. The corresponding screw- and edge- TDDs are estimated to be 6.3 × 10^7^ cm^−2^ and 3.2 × 10^8^ cm^−2^, respectively^13^. The crystalline quality of the AlN films is significantly improved in comparison to our previous work^14^. In addition, our subsequent experiments prove that AlN films with low TDD can be stably and repeatedly acquired from our 15 growth runs using the same epitaxial conditions on the optimized 650/1000 NPSS, with FWHM deviation no more than 5%, which means that a reproducible process for quality control has been achieved for high-quality AlN heteroepitaxy.

**Figure 3:**
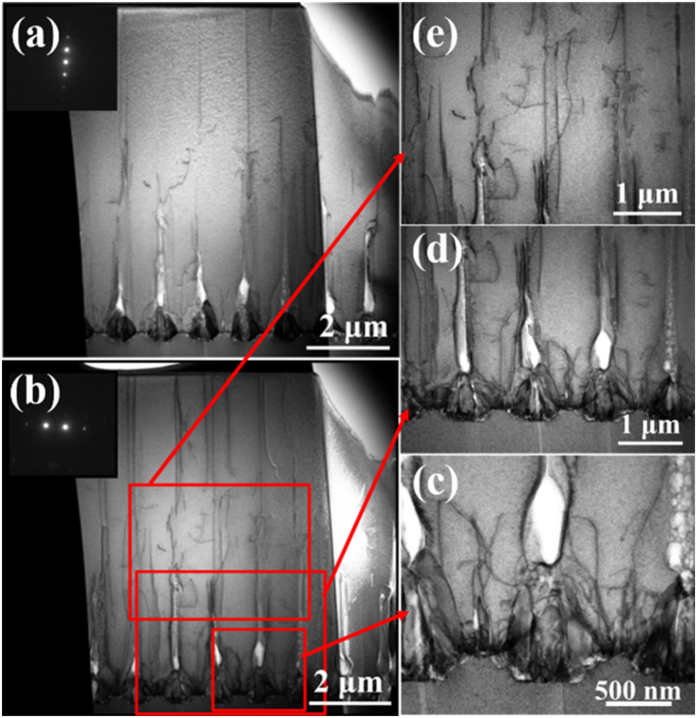
Cross-sectional bright-field STEM images under two-beam conditions for AlN grown on NPSS with (**a**) g = [0002], and (**b**) g = [11
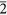
0], (**c–e**) are the magnified STEM images of three selected typical zones in (**b**).

To examine the role of the NPSS pattern in defect control, STEM measurement was further performed to investigate the structural characteristics. Figure 3(a,b) show the cross-sectional bright-field STEM images of an AlN epilayer grown on 650/1000 NPSS at the same area under two-beam condition with **g** = [0002] and **g** = [11
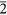
0], respectively. Based on the standard Burgers vector analysis using invisibility criterion **g · b** = 0, the dislocation type can be identified^15^. Screw-type TDs are visible with **g** = [0002], while edge-type TDs are visible with **g** =  <11
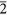
0> . It is evident that many TDs including screw- and edge- types are firstly generated at the AlN/sapphire interface above the un-etched mesa zones. And few of them can thread into the upper AlN epilayer, presenting a similar evolution behavior for both screw- and edge- types TDs. To examine the evolution more clearly, the magnified TEM images of three selected bottom-up zones in Fig. 3(b) for the dominant edge-type TDs are further demonstrated. As shown in Fig. 3(c), almost all TDs in the vicinity of the voids bend towards the void sidewalls and terminate over there due to the image force effect during the lateral overgrowth process, and thus decreasing the TDD above the mesa regions. Since these voids are of cone shape and the scope of the image force is several hundreds of nanometers^16^, it presents a very high efficiency in bending TDs on this optimized NPSS. What’s more, it should be noted that, the magnitude of image force is in inverse proportion of the distance between the void sidewall and the dislocation, so the image force will have a better effect on the dislocations originating from the AlN/sapphire interface when the growth mesa is relatively narrow. These results may shed light on the origins of the FWHM evolution with the hole diameter varying from 300 to 650 nm.

Figure 3(d,e) further focus on illustrating distributions of TDs around the coalescence zones. Apparently, some new TDs appear in these areas during the epitaxial process, implying an increase in the TDD. It is well known that during the ELO process on micro-stripe patterned substrate, different regions merely meet at the ultimate position and the misorientation manifests by incoherent boundaries^4^. Although the boundary effect on NPSS is possibly slight due to nano-scale pattern size compared to the micro-stripe pattern case, it is still inevitable to introduce some TDs due to the misorientation of the adjacent regions above each hole during the coalescence process. When the hole diameter is increased, i.e., the width of the growth mesa is decreased, it will expand the distance between the adjacent regions for this NPSS. In this situation, AlN on the adjacent mesa need to go through a longer distance for both vertical and lateral directions if keeping the epitaxial conditions unchanged, until they meet with each other, and thus increases the possibility of misorientations between these regions. In fact, the *in-situ* reflectance measurement shows an obvious increase of the coalescence thickness when increasing the hole diameter of the NPSS (not shown here). These aggravated misorientations induced by the greater coalescence dimension are believed to account for the increase of FWHM values in Fig. 2 when the hole diameter is increased.

**Figure 4:**
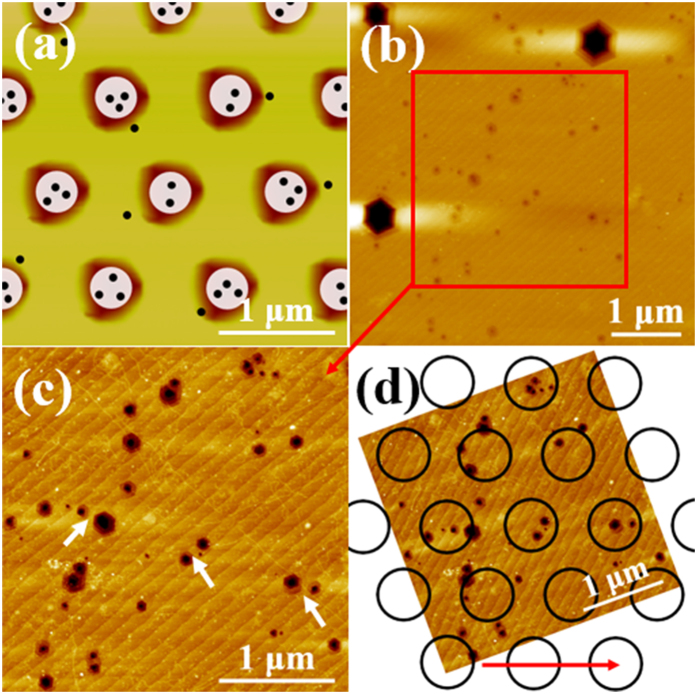
(**a**) The schematic of the dislocation distribution above the NPSS. AFM images of the after-wet-etching AlN (**b**) in 5 × 5 μm^2^, (**c**) in 3 × 3 μm^2^. (**d**) The relationship between the positions of etching pits with the holes patterns on the NPSS.

The distribution of TDs for AlN epitaxy on NPSS is further analyzed for the sake of deeply understanding the STEM images. In Fig. 3(b), 9 or 10 lines are observed near the sample surface (≈5-μm-width). However, it is evident that almost all of the TDs locate above the holes’ region. So a plan view schematic model is proposed in Fig. 4(a) to expound this phenomenon. The white discs represent regions with high TDD and the black dots represent dislocation outcrops. In order to verify the reasonability of the schematic model and STEM images, the AlN grown on NPSS is characterized by wet etching in molten KOH/NaOH under 350 °C for about 90 seconds^17^. The AFM images in Fig. 4(b,c) show the after-etching surface morphologies of AlN. The etching pits density in Fig. 4(b,c) is 2.7–4.2 × 10^8^ cm^−2^ with manual count. The large and small etching pits are usually related with screw- and edge-type dislocations, respectively^18^. It is evident that distribution of TDs is in an un-uniform way. The relative position relationship between Fig. 4(c) and the simplified outlines of holes on the NPSS is determined according to the reference edge of the sapphire, as shown in Fig. 4(d). The red arrow in Fig. 4(d) marks one of the close direction of the holes on the NPSS. As illustrated in Fig. 4(d), most of the TDs distribute in the outlines, which is only 38% of the total area on the substrate. This distribution feature of TDs roughly corresponds to the schematic model. The STEM sample is fabricated along the close direction of holes and close to the centers of holes as much as possible. In this case, the distribution of TDs in the STEM images is similar to the distribution of TDs marked with white arrows in Fig. 4(c).

**Figure 5:**
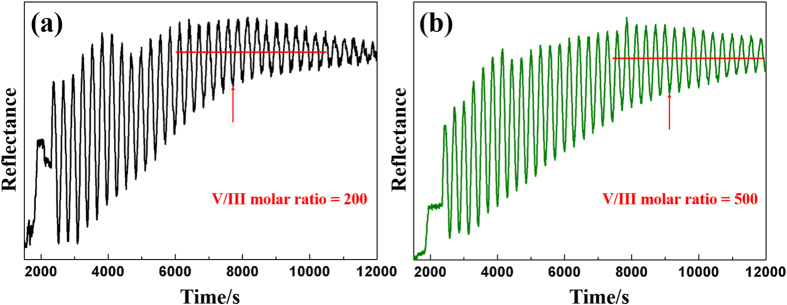
Two typical *in-situ* reflectance lines (405 nm) for different V/III molar ratio conditions on NPSS with the hole diameter of 650 nm, and other conditions remain unchanged. (**a**) Reflectance line of epitaxy under V/III molar ratio of 200. (**b**) Reflectance line of epitaxy under V/III molar ratio of 500.

In addition, it should be stressed that V/III molar ratio is very important in terms of growth kinetic, besides the application of the selected NPSS. Generally, a higher V/III molar ratio leads to a lower lateral growth rate in epitaxial lateral overgrowth process^19^. Figure 5(a,b) show two typical *in-situ* reflectance lines (405 nm) for different V/III molar ratio conditions on NPSS with the hole diameter of 650 nm, and other conditions remain unchanged. Each reflectance oscillation represents a thickness of 90 nm for AlN epitaxy. After preliminary accomplishment of coalescence, reflectance lines begin to oscillate by the same center axis. The red lines in Fig. 5 help us to identify the time of preliminary accomplishment of coalescence and the red arrows mark the positions. The coalescence process experiences 19 and 24.5 periods under the V/III molar ratio of 200 and 500, respectively. It indicates that the coalescence completion is postponed under relatively higher V/III molar ratio, i.e., the lateral growth rate decreases with increasing V/III molar ratio. Since fast growth rate in lateral direction results in tilted growth fronts, leading to coalescence of crystallographically misaligned domains^4^, the increase of the lateral growth rate will lead to aggravated misorientations among different coalescence regions above the hole. Then the TDD will increase at the coalescence region due to the larger degree of misorientation, considering misorientation of boundary will be reflected in the formation of dislocations. So a lower lateral growth rate, i.e., a slightly high V/III molar ratio would decrease the TDD of AlN film. In our experiments, when lower V/III molar ratio is used in later stage of the coalescence process on the optimizes 650/1000 NPSS, i.e., decreasing the V/III molar ratio from 500 (the optimized value) to 200 while keeping the other conditions unchanged, there is an obvious increase in FWHM values for both (0002) (~20 arcsec) and (10
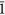
2) (~50 arcsec) peaks compared to the narrowest FWHM values ever shown in Fig. 2, which means a high V/III molar ratio is beneficial to alleviate the issue of misorientations, suggesting that low lateral growth rate is vital for misorientations control in AlN epitaxy on NPSS.

**Figure 6:**
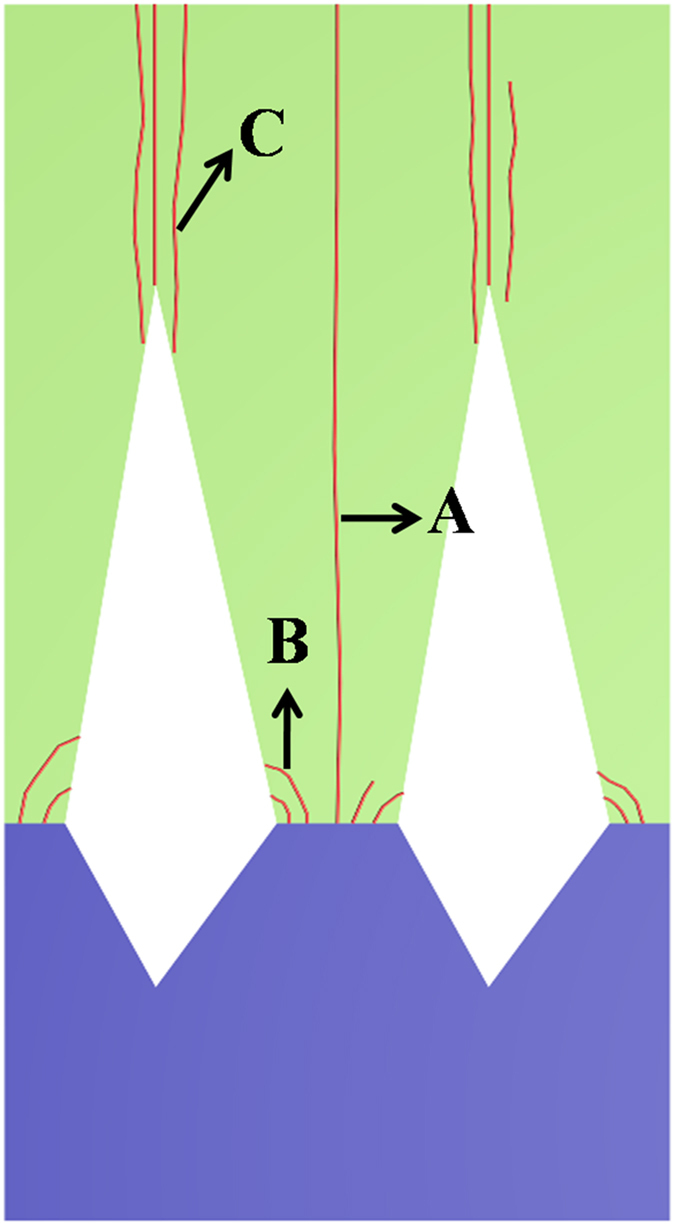
Three main competitive processes influencing TDD of AlN epilayers on NPSS.

Based on the above analyses, it is deduced that there are three main competitive processes influencing TDD in the top AlN epilayers on NPSS, as schematically shown in Fig. 6. The first is process A, where a large number of TDs including screw- and edge- types are generated at AlN/sapphire interface on the un-etched mesas due to the large lattice mismatch. When these TDs propagate vertically up into the top epilayers, there should be a great increase in TDD. The second is process B, where TDs near the voids tend to bend towards the void sidewalls driven by the image force, which can effectively decrease the TDs (screw- and edge- types) on the mesa regions. The third is process C, where some TDs (screw- and edge- types) are generated around the boundaries during coalescence caused by misorientations between the adjacent regions on the mesas. When the hole diameter is increased, i.e., the width of the growth mesa is decreased, TDs from procss A will decrease, and more importantly, process B will gradually become the dominant one that nearly all of the TDs originating from process A can be eliminated via bending from process B with increasing the hole diameter to an optimized value. Whereas with further increasing the hole diameter, the misorietations of process C will gradually become the crucial factor, resulting in deterioration of the cyrstalline quality of AlN. As such, a strategy for decreasing the TDD in the top AlN epilayers on NPSS can be put forward, which means suppressing TDs from process A via process B with the optimized patterns size, and then decreasing TDs from process C. These suggest that, to gain a deeper insight into the role of NPSS in AlN epitaxy, both the effect of image force and the impact of misorientations should be taken into account. Especially, process C mentioned above had better to be accomplished adopting low lateral growth rate across the coalescence process in terms of growth kinetics besides the consideration of the optimized NPSS.

In summary, epitaxial growth of AlN films with atomically flat surface on NPSS has been investigated. The crystalline quality can be greatly improved by using the optimized 650/1000 NPSS achieving the XRD ω-scan FWHM values being 171 and 205 arcsec for (0002) and (10
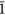
2) reflections, respectively. The optimized D/1000 contributes to eliminate almost entirely the TDs originated from the AlN/sapphire interface via bending the TDs by image force from the void sidewalls before coalescence. In addition, reducing misorientations of the adjacent regions during coalescence adopting low lateral growth rate is also essential for decreasing TDD in the upper AlN epilayer.

## Methods

### Fabrications

**Figure 7:**
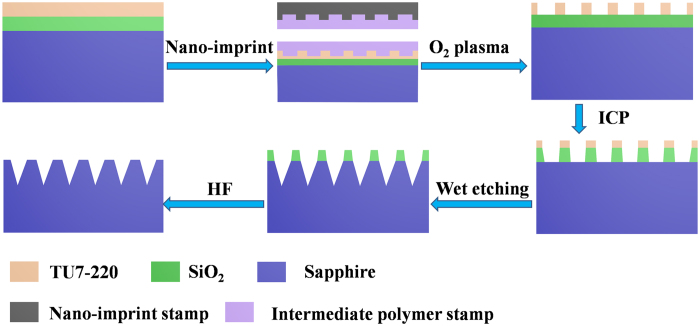
The fabrication process flow to obtain nano-patterned sapphire substrate by nano-imprint lithography.

The fabrication process flow to obtain nano-patterns on a 2-in. c-plane sapphire substrate is shown in Fig. 7. First, a SiO_2_ film is deposited onto a sapphire substrate by plasma-enhanced chemical vapor deposition, and then the nano-imprint resist (NPR) TU7-220 is spin-coated. Second, the hexagonal hole array (hole diameter is 650 nm, and the period is 1000 nm) is transferred to the resist by nano-imprint lithography. A two-step simultaneous thermal and ultraviolet curing (STU) imprint process is applied using an Eitre^®^ 3 nano-imprint instrument. Third, after removing the residual resist at the bottom of the holes by oxygen plasma, SiO_2_ was exposed from the nano holes on the NPR, and the patterns are then transferred to the SiO_2_ film by inductively coupled plasma (ICP) etching. Finally, the sapphire substrate is etched in a mixture of H_2_SO_4_ and H_3_PO_4_ solution for several minutes at 260 °C, and the SiO_2_ mask is removed by HF solution. The depth of holes is about 220 nm for NPSS with the hole diameter of 650 nm when the wet etching process is complete.

All the AlN samples were grown on c-plane NPSS in a 3 × 2″ close-coupled-showerhead (CCS) Aixtron MOCVD system. Trimethylaluminum (TMAl) and ammonia (NH_3_) were used as the precursors. H_2_ was the carrier gas. *In-situ* reflectance measurements at 405 nm were carried out using a LayTec EpiTT system to record the growth oscillations. The growth conditions are optimized from the following four aspects: temperature, pressure, TMAl flow and V/III molar ratio. In order to overcome high diffusion barrier of Al adatoms during AlN epitaxy, almost the maximum temperature (1250 °C) that our MOCVD can handle is applied as the epitaxial temperature, and a low pressure is used to suppress the parasitic reaction. Then the XRD FWHM values are taken as the prior evaluation index to select the optimized epitaxial conditions by orthogonal experiment for about 100 runs, mainly concerning TMAl flow (50~80 sccm) and V/III molar ratio (100~600). Finally, it is found that crystalline quality of AlN epitaxy on NPSS is sensitive to V/III molar ratio, while insensitive to the TMAl flow. For all the samples, the deposition was initiated with a 25-nm-thick AlN nucleation layer at 950 °C. Then the temperature was increased to 1250 °C for growing a 6-μm-thick high temperature (HT) AlN layer under 50 mbar with the optimized V/III molar ratio of 500. During the wet etching, the AlN grown on NPSS is characterized by molten KOH/NaOH under 350 °C for about 90 seconds^17^.

### Measurements

Cross-sectional scan transmission electron microscope (STEM) is used to analyze the dislocations evolution process during epitaxy, the sample is fabricated by focused ion beam (FIB) and the thickness is about 100 nm. High-resolution X-ray diffraction (HRXRD) is used to evaluate the crystalline quality of the AlN films. Atomic force microscope (AFM) is used to study the surface morphologies of the NPSS and AlN films, and the High Aspect Ratio probe is used to measure the wet etching AlN.

## Additional Information

**How to cite this article**: Zhang, L. *et al*. High-quality AlN epitaxy on nano-patterned sapphire substrates prepared by nano-imprint lithography. *Sci. Rep.* **6**, 35934; doi: 10.1038/srep35934 (2016).

**Publisher’s note:** Springer Nature remains neutral with regard to jurisdictional claims in published maps and institutional affiliations.
